# Impact of Prosthodontic Treatment on the Oral Health-related Quality of Life in Mucormycosis Patient: A Case Report

**DOI:** 10.7759/cureus.3493

**Published:** 2018-10-24

**Authors:** Athiban Inbarajan, Shanmuganathan Natarajan, Saravanan Thirumalai Thangarajan, Madhankumar Seenivasan, Fathima Banu, V Anand Kumar

**Affiliations:** 1 Prosthodontics, Sri Ramachandra University, Chennai, IND

**Keywords:** mucormycosis, prosthodontic rehabilitation, complete dentures, likert’s scale, oral health-related quality of life

## Abstract

Mucormycosis is a fulminant fungal infection that occurs most often in diabetic and immunocompromised patients, including those with hematologic malignancies. Patients with maxillary resections present a challenge situation for the maxillofacial prosthodontist. Prosthodontic rehabilitation of such patients presents a significant challenge in restoring speech, deglutition and mastication, and respiration. This report discusses the impact of post-surgical management of mucormycosis patient with prosthodontic treatment and evaluating the oral health-related quality of life using the Oral Health Impact Profile-14 (OHIP-14) questionnaire.

## Introduction

According to the World Health Organization (WHO), “health is a state of complete physical, mental, and social well-being and not merely the absence of disease or infirmity.” Of the three dimensions in quality of life (QoL) (namely, social, political, and health) [1], health-related quality of life (HR-QoL) relates to optimum levels of mental, physical, and social well-being. Oral health-related quality of life (OHR-QoL) is that part of life affected by oral functions, psychological factors, and pain/discomfort experienced in the oro-facial zone [2]. Mucormycosis or phycomycosis is a head and neck infection caused by the organism Mucorales belonging to the class Phycomycetes, one of the most rapidly fatal fulminate mycotic infections in human beings [3], which leads to necrosis and destruction of the involved structures and eventually results in decreased HR-QoL. This is usually seen in patients with predisposing debilitating conditions, like diabetes mellitus, blood dyscariasis, neutropenia, and patients under immunosuppressive therapy [3]. The management of such cases is by complete surgical debridement with total or subtotal maxillectomy. Rehabilitation of maxillectomy defects aims to restore the lost functional, as well as psychological, factors, thereby attempting to improve the OHR QoL. The Oral Health Impact Profile-14 (OHIP-14) is a self-filled questionnaire that describes the impact of oral health conditions in seven domains, namely functional limitation, physical pain, psychological discomfort, physical disability, psychological disability, social disability, and handicap [4]. The responses are recorded on a 5-point Likert’s scale ( 0 - never, 1 - hardly ever, 2 - occasionally, 3 - fairly often, and 4 - very often), with "never" indicating the least impact and "very often" indicating the maximum impact. It is the short form of the original extended version of 49-items developed by WHO [5]. In spite of being a short-questionnaire, it has proved to be reliable with adequate cross-cultural consistency and sensitive to changes [6]. Maxillectomy results in a functional and psychological deficit, so it is a challenge to the prosthodontist to restore and rehabilitate the lost function and psychological balance.

The aim of this clinical report is that it describes the prosthodontic rehabilitation of the maxillectomy defect of a patient treated for mucormycosis, followed by evaluating the oral health-related quality of life using the OHIP-14 questionnaire.

## Case presentation

A 60-year-old female patient, status-post maxillectomy for a case of mucormycosis, reported to the Department of Prosthodontics with the chief complaint of missing teeth in the maxillary and mandibular arch that affected her aesthetics and masticatory function. The patient also complained of nasal regurgitation of food and hypernasality of voice. At the time of presentation, the patient was demoralized and psychologically unstable. The maxillectomy adversely affected the patient’s psychological state as she failed to communicate and follow commands, along with unintelligible speech.

**Figure 1:**
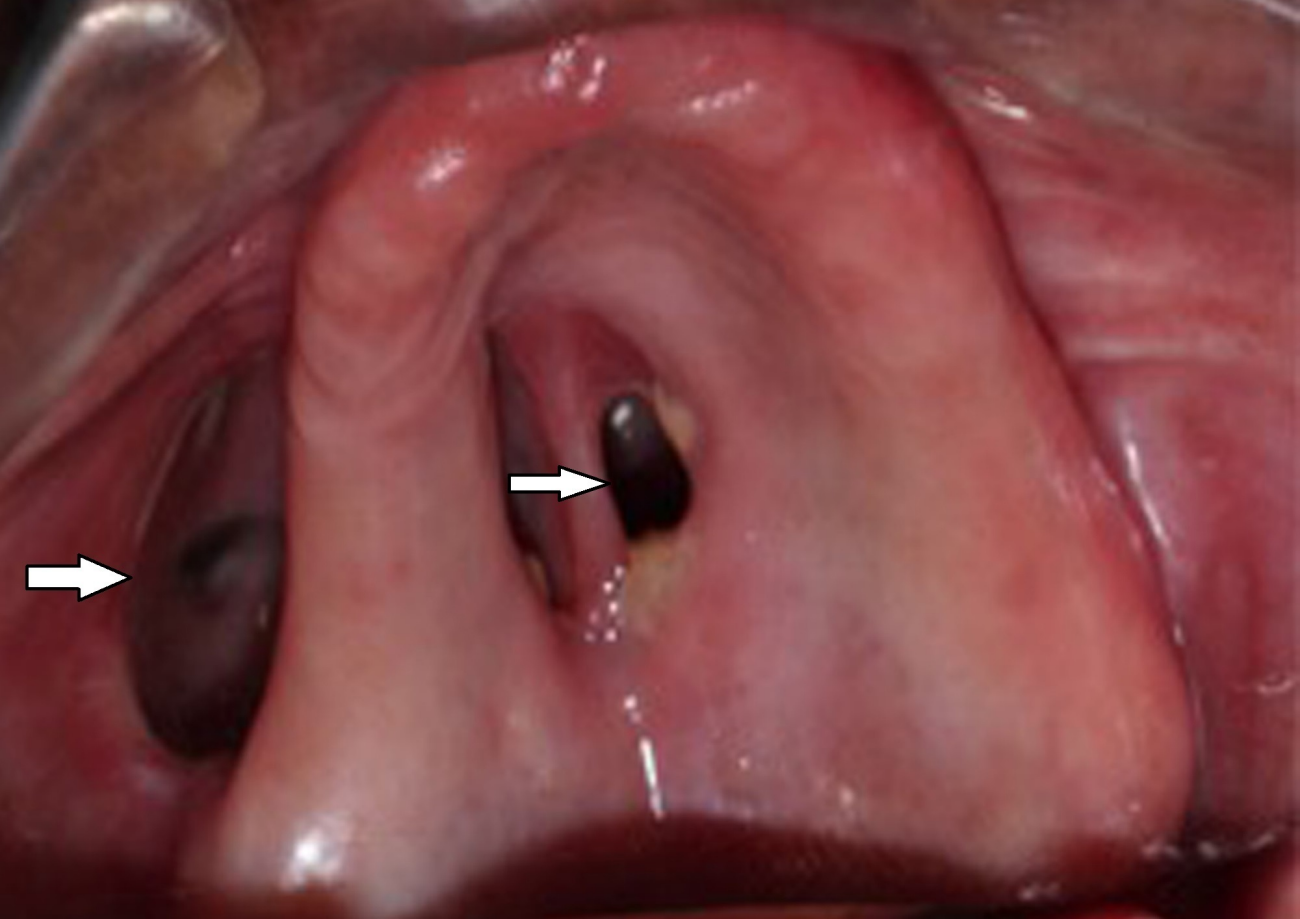
Post-surgical intraoral defect in the maxilla

On extraoral examination, facial asymmetry was found. Her past medical history revealed that the patient had uncontrolled type II diabetes mellitus for which she had been on medication for the past 15 years. The patient was malnourished and was taking nutritional supplements for the same. The patient was diagnosed with mucormycosis of the left maxillary sinus a year earlier for which she underwent surgical debridement one month prior to presentation. Intraoral examination revealed a completely edentulous maxilla and mandible and an oronasal fistula on the left side of the maxilla. The oral side of the defect extended into the buccal vestibule and lateral to the left hard palate with an adequate amount of alveolar ridge overlying the defect (Figure 1).

Prosthodontic intervention

**Figure 2:**
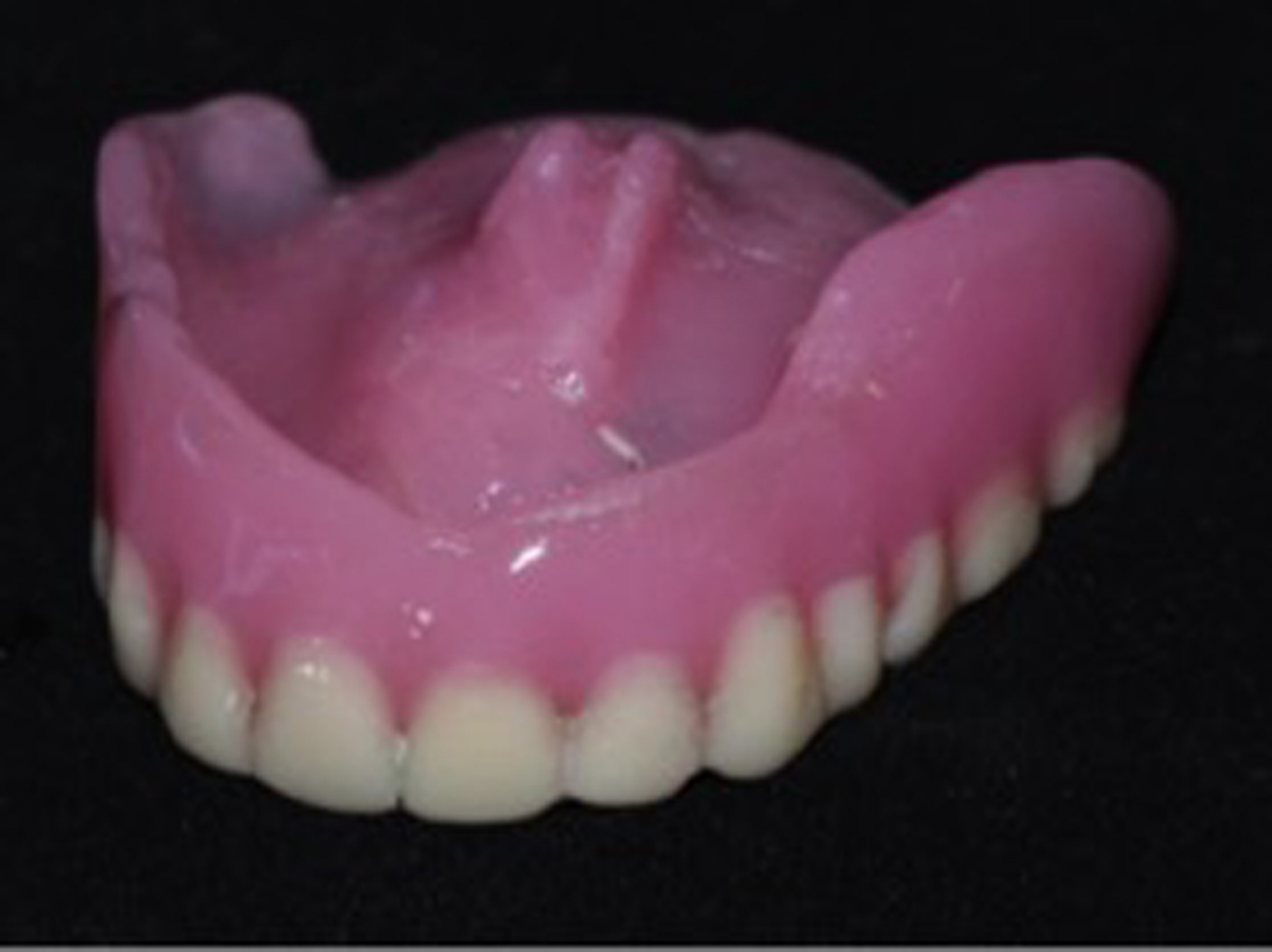
Maxillary complete denture showing the coverage of the defect

After discussing the possible treatment options with the patient, considering her age and medical condition, fabrication of a conventional complete denture prosthesis that would restore aesthetics and function, as well as obliteration of the fistula, was planned. A maxillary and mandibular preliminary impression was taken with irreversible hydrocolloid using a stock tray (after packing the defect with gauze to prevent impression material from entering the nasal cavity). An impression was poured with dental stone Type IV and a custom tray was fabricated using auto polymerizing acrylic resin. Border molding was done with green stick compound, and a second impression was made with elastomeric impression material after blocking the defect with gauze. In the master cast, the vestibular and palatal defect was blocked with wax to relieve undercuts before fabrication of the denture base and occlusal rim. Tentative jaw relation was done and transferred to a mean value articulator for artificial teeth arrangement. During the wax try-in appointment, the patient's centric relation, esthetics, and phonetics were assessed. A functional impression was made using the trial denture maintaining the correct vertical dimension at occlusion. It was then processed with heat-cured acrylic denture base material (Figure 2).

**Figure 3:**
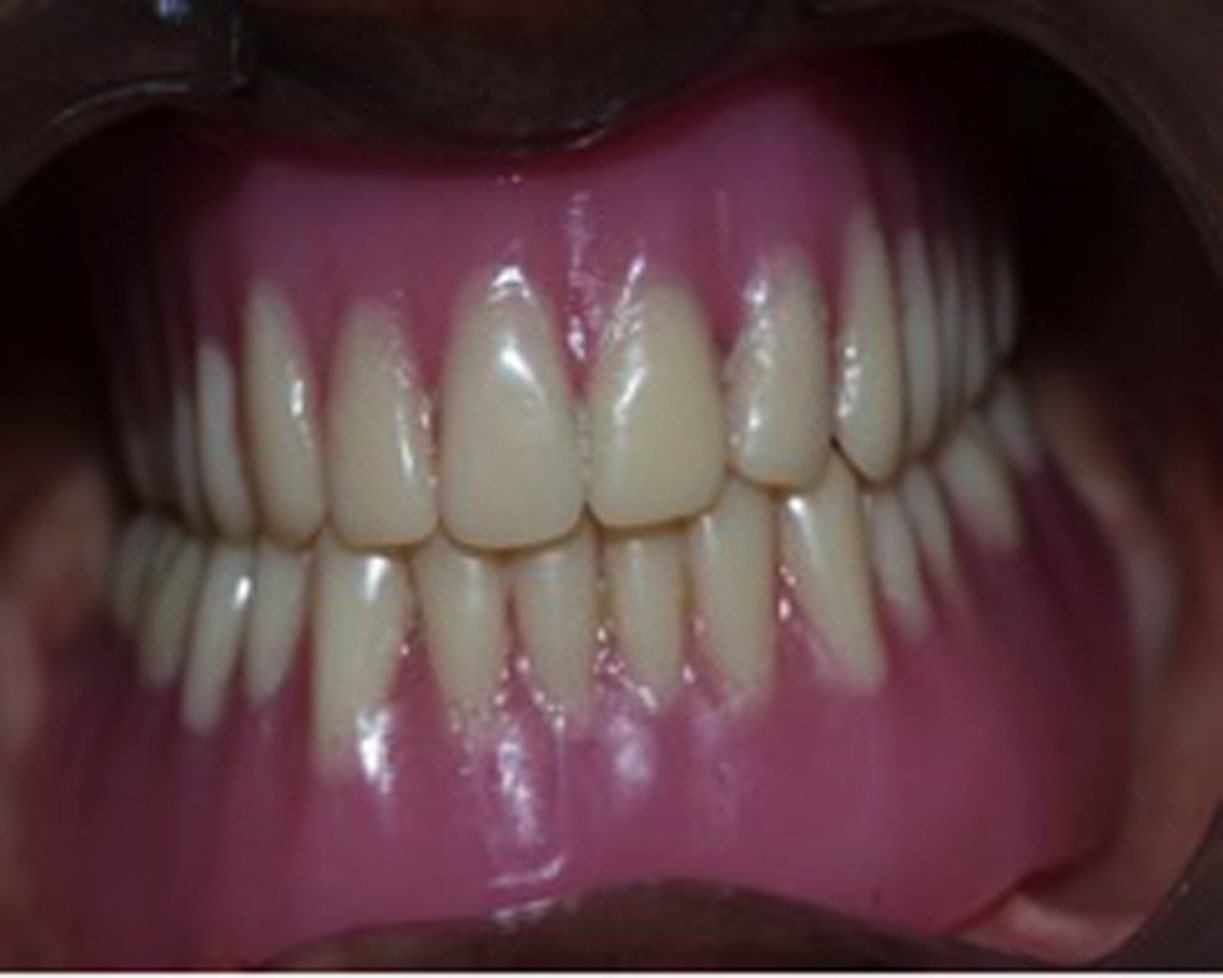
Complete denture in occlusion

During the denture insertion appointment, the patient's occlusion, phonetics, and esthetics were checked, and the patient was asked to drink water to check for nasal regurgitation of fluids (Figure 3).

**Figure 4:**
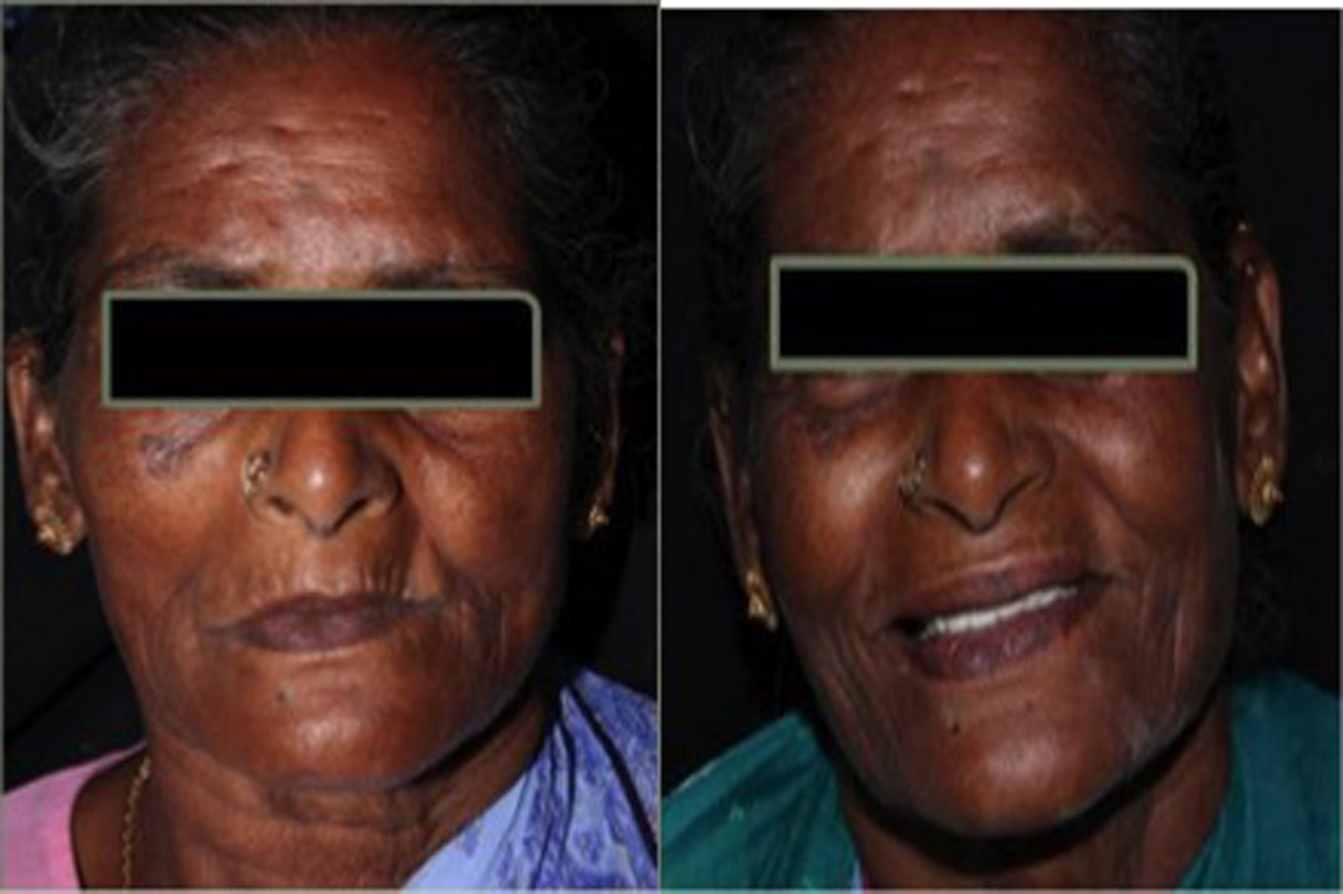
Patient before and after the treatment

Post-insertion instructions on proper use, care, and maintenance of the prosthesis were given to the patient. The patient was reviewed after 24 hours and once every two weeks for the next three months (Figure 4).

During the follow-up visits, the patient was reevaluated for minor adjustments. The OHIP-14 questionnaire was evaluated pre- and post-complete denture treatment. The details of the treatment were explained, and informed consent was taken from both the patient and the patient’s attender, as the patient was depressed and psychologically unstable.

At the end of three months, a second OHIP-14 questionnaire was recorded. The OHIP-14 questionnaire was used to assess the impact of prosthodontic treatment on the OHR-QoL of the patient. The pre- and post-treatment responses of the OHIP-14 questionnaire are tabulated (Table 1) [4, 6].

**Table 1:** Pre- and Post-treatment Responses of OHIP-14 The responses were recorded on a 5-point Likert’s scale (0 - never, 1 - hardly ever, 2 - occasionally, 3 - fairly often, and 4 - very often). OHIP-14: Oral Health Impact Profile-14

Dimensions	Question	Pre-treatment Response	Post-treatment Response
Functional Limitation	Have you had trouble pronouncing any words because of problems with your teeth, mouth, or dentures?	4	1
	Have you felt that your sense of taste has worsened because of problems with your teeth, mouth, or dentures?	4	1
Physical pain	Have you had painful aching in your mouth?	3	0
	Have you found it uncomfortable to eat any foods because of problems with your teeth, mouth, or dentures?	3	1
Psychological Discomfort	Have you been self-conscious because of your teeth, mouth, or dentures?	3	1
	Have you felt tense because of problems with your teeth, mouth, or dentures?	4	2
Physical Disability	Has your diet been unsatisfactory because of problems with your teeth, mouth, or dentures?	4	1
	Have you had to interrupt meals because of problems with your teeth, mouth, or dentures?	3	1
Psychological Disability	Have you found it difficult to relax because of problems with your teeth, mouth, or dentures?	4	1
	Have you been a bit embarrassed because of problems with your teeth, mouth, or dentures?	4	1
Social Disability	Have you been a bit irritable with other people because of problems with your teeth, mouth, or dentures?	3	0
	Have you had difficulty doing your usual jobs because of problems with your teeth, mouth, or dentures?	4	0
Handicap	Have you felt that life, in general, was less satisfying because of problems with your teeth, mouth, or dentures?	4	1
	Have you been totally unable to function because of problems with your teeth, mouth, or dentures?	3	0

## Discussion

One form of mucormycosis is called rhinocerebral mucormycosis, which typically targets the maxillary antrum and invades the surrounding tissue, causing a blackish slough of necrotizing ulceration of the palate with exposure of underlying bone. These patients usually present with facial pain, nasal discharge, and sinusitis with clinical signs of orbital cellulitis and necrotic black tissue in the nasal turbinates and septum. As the condition progresses, patients suffer from metabolic derangements, such as liver/renal failure and uncontrolled diabetes with ketoacidosis, later with a confused state of mind and then may slip into a coma. Aggressive surgical debridement is commonly instituted, which results in the loss of the palate, maxilla, and contiguous structures, followed by difficulties with speech, deglutition, mastication, and respiration. Even with recent advances in diagnosis and treatment, a high mortality of 30 - 70% has been documented in the literature with no age or gender predilection. An obturator prosthesis is the treatment of choice because it creates a partition between the oral and nasal cavities, restores facial contour, improves mastication, articulation and speech intelligibility, and provides lip support. Support and retention of the prosthesis are often difficult to achieve due to the absence of teeth, a lack of favorable undercuts, and the presence of non-keratinized nasal mucosa.

Impairment of senses, such as taste, smell, and hearing, along with functional disabilities and compromised esthetics, promotes a negative impact on the patient’s OHR-QoL [7]. Psychological depression is the final outcome of life stresses like medical illness [8]. Maxillectomy adversely affects not only function and aesthetics but also results in emotional disturbances [9]. As stated, the above factors could be the possible reason for depression in the case presented. Under the following circumstances, an immediate prosthesis becomes a necessity to lessen the psychological impact. Postoperatively, the obturator was delivered after a month due to delayed reporting of the patient for prosthetic rehabilitation, thus adding to the patient’s psychological distress. Hence, the trauma from the surgery, her edentulous condition, and impaired function, along with the delayed prosthesis, were the possible factors for the patient’s distress and anxiety [8-9]. There was improved speech post-treatment, which was due to the better stability of the prosthesis [7]. A good denture also provides improved masticatory efficiency that leads to improved taste perception.

The post-treatment responses were recorded after three months. There was a gradual shift in the OHIP-14 from the right side to the left side in the Likert’s scale between pre- and post-treatment responses. Improved post-treatment impact in the OHR-QoL could be due to several reasons, such as elaborative counseling, improved prosthesis stability, masticatory efficiency along with taste, and the patient’s enhanced mental health. It is required to record similar responses at a later period, so further follow-up appointments were scheduled with the anticipation of further improvement of OHR-QoL.

Thus, a prosthodontist plays a vital role in the rehabilitation of total/subtotal maxillectomy patients by separating the oral and the nasal cavities, restoring the normal speech and mastication, along with improved aesthetics. In the presented case, the emphasis was more on the intaglio surface and occlusion with reduced cuspal angles for better retention and stability of the prosthesis functionally. Though an acrylic prosthesis was fabricated, metal could have been a better option; however, owing to the cost factor and the increase in the number of visits, acrylic was preferred. An implant-supported prosthesis was a viable treatment option for better retention, but conventional obturators were preferred to avoid further surgical intervention. Several studies have reported the successful rehabilitation of maxillectomy patients with conventional obturators.

## Conclusions

The major role of a prosthodontist in treating maxillectomy patients does not end by rehabilitation of the lost oral function and aesthetics but also by rehabilitating and restoring the patient’s mental health. Early diagnosis, eradication of the predisposing factor, and surgical debridement, along with antifungal therapy, are of prime importance for successful treatment and patient survival. Although the difference in pre- and post-treatment responses was minimal owing to the sample size and duration, it definitely showed a positive impact on the patient's mental health, thereby improving her oral health-related quality of life. Hence, further studies with a good sample size and duration are required for further validation.
